# Inattentive Driving Detection Using Body-Worn Sensors: Feasibility Study

**DOI:** 10.3390/s22010352

**Published:** 2022-01-04

**Authors:** Takuma Akiduki, Jun Nagasawa, Zhong Zhang, Yuto Omae, Toshiya Arakawa, Hirotaka Takahashi

**Affiliations:** 1 Graduate School of Engineering, Toyohashi University of Technology, Toyohashi 441-8580, Japan; nagasawa@is.me.tut.ac.jp; 2 Department of Intelligent Mechanical Engineering, Hiroshima Institute of Technology, Saeki-ku, Hiroshima 731-5193, Japan; t.sho.g4@cc.it-hiroshima.ac.jp; 3 Department of Industrial Engineering and Management, College of Industrial Technology, Nihon University, Narashino 275-8575, Japan; oomae.yuuto@nihon-u.ac.jp; 4 Department of Information Technology and Media Design, Nippon Institute of Technology, Miyashiro-machi, Saitama 345-8501, Japan; arakawa.toshiya@nit.ac.jp; 5 Research Center for Space Science, Advanced Research Laboratories, Tokyo City University, Setagaya-ku, Tokyo 158-0082, Japan; hirotaka@tcu.ac.jp

**Keywords:** accelerometer, motion feature, body-worn sensor, drowsiness driving, inattentive driving

## Abstract

This study aims to build a system for detecting a driver’s internal state using body-worn sensors. Our system is intended to detect inattentive driving that occurs during long-term driving on a monotonous road, such as a high-way road. The inattentive state of a driver in this study is an absent-minded state caused by a decrease in driver vigilance levels due to fatigue or drowsiness. However, it is difficult to clearly define these inattentive states because it is difficult for the driver to recognize when they fall into an absent-minded state. To address this problem and achieve our goal, we have proposed a detection algorithm for inattentive driving that not only uses a heart rate sensor, but also uses body-worn inertial sensors, which have the potential to detect driver behavior more accurately and at a much lower cost. The proposed method combines three detection models: body movement, drowsiness, and inattention detection, based on an anomaly detection algorithm. Furthermore, we have verified the accuracy of the algorithm with the experimental data for five participants that were measured in long-term and monotonous driving scenarios by using a driving simulator. The results indicate that our approach can detect both the inattentive and drowsiness states of drivers using signals from both the heart rate sensor and accelerometers placed on wrists.

## 1. Introduction

Inattentive driving is one of the leading causes of traffic accidents, accounting for approximately 20% of fatal accidents in Japan [1]. The inattentive state of a driver in this study is an absent-minded state caused by a decrease in driver vigilance levels due to fatigue or drowsiness. Thus, a driver assistance system for monitoring these drivers’ internal states, such as the driver’s vigilance or drowsiness, is important to prevent such accidents.

Inattention detection systems can be classified into two categories according to the types of their input signals. One category uses vehicle signals such as the speed and steering angle [2,3,4,5,6,7]. Kume et al. [3] reported that the sensitivity of detecting an inattentive state using in-vehicle sensors was 53% with a fixed threshold, and 82% with a manually set threshold. However, it is difficult to detect weak drowsiness that the driver is unaware of from vehicle signals [8].

The other category is the use of physiological and behavioral signals from a driver that can directly capture changes in the driver’s internal state. The major measurements include eye and facial movements [8,9,10,11,12,13,14], heart rate variability [15,16,17], and brain activities [18,19]. Algorithms based on image processing can detect eye and facial movements with non-contact; however, it is difficult to stably measure the eye and facial movements during the day or night because image processing is affected by the level of illumination. For detection methods using an electroencephalogram (EEG), it is necessary to attach electrodes to the head of the driver, which is impractical for everyday use in a real car environment.

In their methods, it is difficult to stably measure the fine changes in the signals due to sleepiness and the detection accuracy varies greatly among individuals. Therefore, there exists a need for more stable measures to improve detection accuracy, such as the combination of biological and behavioral indicators. As a behavioral indicator, the driver’s body movements, called subsidiary behaviors, which are not directly related to driving operations, have been investigated. Roge et al. observed the driver’s behavior during long-term monotonous driving tasks on a driving simulator and suggested a relationship between the frequency of occurrence of subsidiary behaviors and the arousal level [20]. Matsuo and Abdelaziz proposed a method to estimate the sleepiness level via detection of the eye closure rate, swaying of the head, and frequency of subsidiary behaviors based on image-processing [21]. Sunagawa et al. also proposed using physiological signals, such as electrocardiogram (ECG) or heart rate analysis, and the change in the driver’s posture, as indicators of the change in sleepiness level [22]. However, these methods require cameras or in-vehicle seat sensors to detect driver behavior. On the other hand, wearable sensors, such as wrist-worn accelerometers, have the potential to detect driver behavior more accurately and at a much lower cost [23,24,25,26].

In our previous studies, Tsubowa et al. [27] measured the frequency of subsidiary behaviors using wrist-worn sensors, including not only yawning and head swaying, but also arm and hand activities, associated with changes in sleepiness level. Then, the feasibility of driver monitoring via wrist-worn sensors was discussed. Additionally, Nagasawa et al. [28] measured the body movement of a car driver using three-axis accelerometers for detecting features of an inattentive driving state. The results suggested that the variance of the acceleration data on the wrists may be reflecting a change in a driver’s internal state; thus, drowsiness and the inattentive state of a driver may be detected by analyzing both physiological and behavioral signals, which can be measured by devices, such as a heart rate monitor or an accelerometer built in a smartwatch.

**Figure 1:**
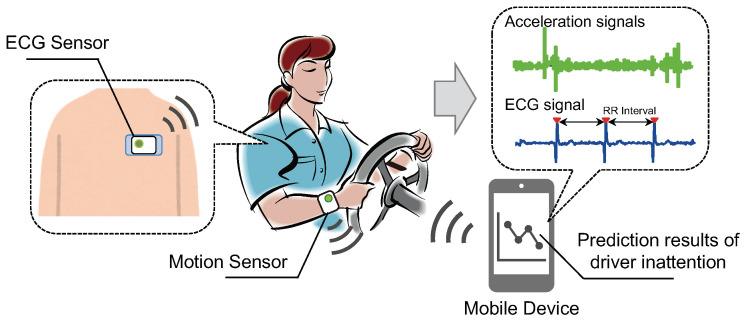
The overview of the proposed system.

This study presents a new detection algorithm for inattentive driving that not only uses a heart rate sensor, but also uses body-worn inertial sensors, based on our previous studies. In our method, an anomaly detection algorithm is applied. In other words, it is an unsupervised method, and the learning procedure is different from supervised binary classification, that is, the data with a normal state only is used for model construction. Furthermore, we have verified the accuracy of the algorithm using experimental data measured by a driving simulator, where five participants were assigned. The driving scenario was designed for collecting the two types of data; one is the condition that simulates normal driving, and another is the condition for inducing a decrease in driver’s performance. The proposed method has the advantage of being implementable in a smartphone or a small wearable device such as that shown in Figure 1, without requiring in-vehicle sensors or a camera.

## 2. Overview of the Proposed System

The overview of our proposed system for detecting inattentive driving is shown in Figure 1. It consists of three parts: data acquisition using body-worn sensors, feature extraction, and internal state detection. In addition, the detection part includes three types of detection: body movement, drowsiness, and inattention.

### 2.1. Detection Algorithm

Inattentive driving is defined as a state in which driving performance is low due to a decrease in the level of vigilance. Vigilance is different from the concept of drowsiness, which refers to a person’s tendency to fall asleep [18]. Unlike a drowsiness state, it is difficult for us to judge the inattentive state from a driver’s facial expression. In addition, it is difficult to recognize the moment when driver himself falls into the inattentive state. For these reasons, measuring the actual state of a driver remains difficult. Therefore, our algorithm is constructed on the basis of the approach of anomaly detection; the detection model is constructed using data from the normal driving state, and a decrease in the vigilance is detected by the degree of the dissimilarity from the normal driving state.

**Figure 2:**
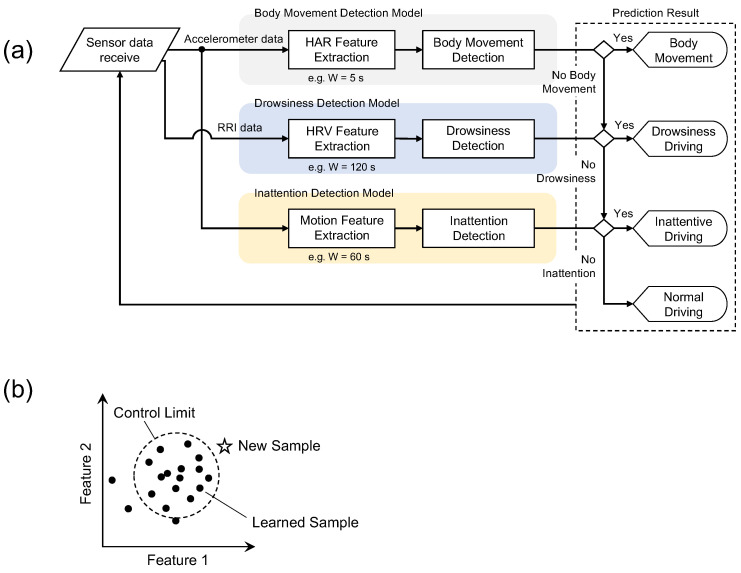
Prediction algorithm in this study: (**a**) flowchart of driver state discrimination; (**b**) schematic diagram for each detection model based on anomaly detection algorithm.

As shown in Figure 2a, the algorithm consists of three detection models; body movement, drowsiness, and inattention; each model performs in parallel. The reason for constructing the three independent models is the difference in the timescale of each phenomenon. For example, the changes in drowsiness are on the order of minutes, while the changes in body movements and the decrease in vigilance are of the order of seconds. Thus, the length of the signal used for the input to each model, called a *window size*, is different for each model. The window moves over sensor data at specified intervals called a *sliding interval*, and each feature is computed over the data in the window.

Then, features are calculated from the sensor data for each sliding interval and set as input to each model. From the detection results of each model, one state is finally determined according to the flow in Figure 2a. The role and function of each model are as follows:

- **Body Movement Detection:** Based on the anomaly detection approach, this model detects wrist movements during monotonous steering operation as a non-anomaly (i.e., “no body movement’’) and other large body movements such as changing the hand position or releasing the steering wheel as an anomaly. Features of wrist movements such as the mean and variance of acceleration, which are widely used in Human Activity Recognition (HAR) [29,30,31], are computed from accelerometer data and used as input to the model. If “no body movement” is determined, the next model determines the state.
- **Drowsiness Detection:** Based on the anomaly detection approach, this model detects a state with an ordinary arousal level as a non-anomaly (i.e., “no drowsiness”), and a decrease in arousal from the normal state is considered an anomaly. Features of heart rate variability (HRV), widely used as an indicator of autonomic nervous system activity, are computed from RR interval (RRI) data and used as input to the model. The RR interval is the time interval of the R wave, which is the positive peak included in the ECG waveform, as shown in Figure 1. If “no drowsiness” is determined, the next model determines the state.
- **Inattention Detection:** Based on the anomaly detection approach, this model detects wrist movements during monotonous steering operation as a non-anomaly (i.e., “no inattention”), and wrist movements during monotonous steering operation in a state of reduced vigilance are considered an anomaly. Motion features described in the below section are computed from accelerometer data to obtain the fine-grained changes of the wrist movement and used as input in the model. If “no inattention” is determined, the state is defined as “normal state”.

As shown in Figure 2b, the “anomaly” in each model is determined by measuring the distance between an input sample and the mean of the learned samples used to model construction. Each model is constructed using a multivariate statistical process control (MSPC) described in Section 2.3, one of the anomaly detection algorithms. In this study, however, we show the results of verifying the two detection models separately, excluding the body movement detection model as a feasibility study.

### 2.2. Feature Extraction

To detect the drowsiness and inattentive driving, two types of features are extracted from RRI data and accelerometer data using the following procedures.

#### 2.2.1. Heart Rate Variability Features

Heart rate variability (HRV) features are calculated according to [15,32]. The following five features in the time domain are directly calculated from the raw RRI data; mean value of RR intervals (meanNN) [ms], standard deviation of RRI (SDNN) [ms], root mean square of successive RRI differences (RMSSD) [ms], total power [ms^2^], and the number of adjacent RRIs that differ from each other by more than 50 ms (NN50). In addition, the following three features in the frequency domain are obtained through the power spectrum density (PSD) of the resampled RRI data, and the PSD is calculated using an autoregressive model; Low Frequency (LF) [ms^2^] (0.04–0.15 Hz), High Frequency (HF) [ms^2^] (0.15–0.4 Hz), and LF/HF [%].

#### 2.2.2. Motion Features

On monotonous roads such as highways, we had to maintain continuous and delicate steering operations to prevent meandering vehicles due to road gradients or crosswinds. However, when driver attention is reduced due to fatigue or low arousal, there is a delay in recognizing the vehicle’s deviation from the lane. Then, there is a delay in driving operation, and the temporal change in both vehicle speed and steering angle becomes small and slow in an inattentive driving state [3,33]. These disturbances in the driving operation are also considered to be reflected in the driver’s limb movement. Therefore, to emphasize and detect these changes from the wrists movements, we define the time difference of the acceleration signals measured at the wrists in the following procedure:

1. Consider a subsequence X extracted by the sliding window [34] of length *W* [s] from the acceleration signal of αth-axis.
2. The subsequence X is divided into $N_{\mathrm{sw}}$ sub-windows and calculated by taking the difference in average amplitude between the adjacent sub-window:
  (1) $$d_{i}^{\alpha }=A_{i+1}^{\alpha }-A_{i}^{\alpha },\;i=1,2,\cdots ,N_{\mathrm{sw}}-1,$$
  where $A_{i}$ is the average amplitude of the acceleration signal at the *i*th sub-window.
3. Calculate a histogram for the $d_{i}^{\alpha }$, and its statistics are used as the *motion features*. In this study, we use three statistics: variance, skewness, and kurtosis to describe the distribution shape. Note that the three motion features, variance/skewness/kurtosis of the difference values within the subsequence, were defined experimentally, which provided the best overall estimation.

By calculating the difference between the averaged values for each region, the changes in the movement of the wrists can be captured regardless of vehicle vibration or steering position.

**Figure 3:**
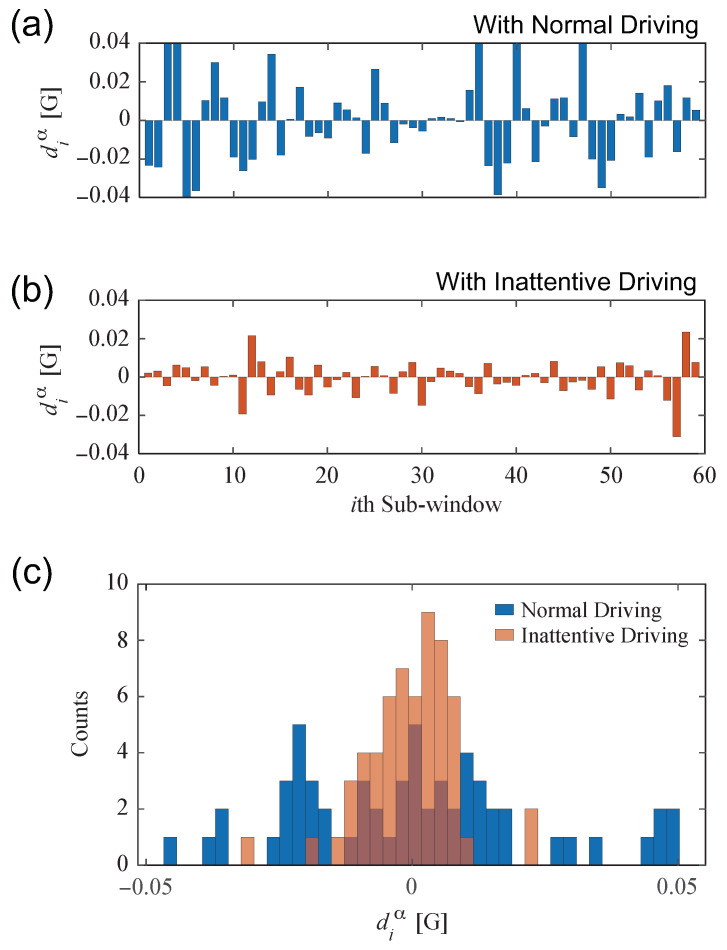
An example of motion features: (**a**,**b**) the difference value $d_{i}^{\alpha }$ corresponding to the normal driving and inattentive driving; (**c**) the histograms of the difference value for (**a**,**b**).

Figure 3a,b shows an example of the dynamic change obtained by dividing the Y-axis acceleration data measured on the left wrist into $N_{\mathrm{sw}}=60$ equally over for a window length of W=60 s. In Figure 3a, fine-grained movements associated with steering operation can be seen, but in (b), the change in motion is smaller than in (a). Note that (b) is a state of inattentive driving based on the reaction time measured by the Vigilance task; the reaction time (RT) in (b) is longer than (a) (RT = 528.5 ms >352.5 ms). These trends might consider that the variation in the difference value became smaller because the awareness of driving decreased and the change in the movement of the wrist became smaller. Figure 3c shows the result of overlaying the difference values of (a) and (b) in a histogram. In the normal driving of (a), the difference value is large, so the frequency distribution is wide, and in the inattentive driving of (b), the change in body movement is small, and the tendency to concentrate is near zero.

### 2.3. Multivariate Statistical Process Control Model

MSPC has been used for monitoring the multivariate process in the field of process control, medical diagnosis, and other numerous processes [15,35,36]. The MSPC models the correlation among variables with principal component analysis (PCA).

Here, consider a data matrix $X\in R^{N\times P}$ whose *i*th row is the *i*th feature vector $x_{i}\in R^{P}$, where *P* and *N* represent the number of variables and samples, respectively. Note that each variable is assumed to be standardized. The singular value decomposition of the data matrix X is written as follows:

(2) $$\begin{array}{cc} X & ={\mathrm{USV}}^{⊤}\\ \; & =\left[\begin{array}{cc} U_{R} & U_{0} \end{array}\right]\left[\begin{array}{cc} S_{R} & 0\\ 0 & S_{0} \end{array}\right]{\left[\begin{array}{cc} V_{R} & V_{0} \end{array}\right]}^{⊤}, \end{array}$$

where U and V are orthogonal matrices, and *R* (≤P) denotes the number of principal components to be retained in a PCA model and $A^{⊤}$ is the transpose of A. The diagonal matrix S has singular values $s_{r}$ in its diagonal elements in decreasing order. From Equation (2), the score matrix is given by,

(3) $$T_{R}=XV_{R}=U_{R}S_{R}.$$

Using Equation (3), *P*-dimensional data can be projected onto the *R*-dimensional subspace. In addition, Hotelling’s $T^{2}$ statistics are used to monitor anomalies on the subspace spanned by principal components:

(4) $$T^{2}=xV_{R}S_{R}^{-2}V_{R}^{⊤}x^{⊤},$$

where $x\in R^{P}$ is a newly measured sample. When the $T^{2}$ statistics calculated for the sample x exceeds the *control limit*, the sample x is determined as an anomaly.

## 3. Experiment

Accelerometer data and RRI data were collected through experiments using a driving simulator to build and validate the detection models in Section 2.1.

### 3.1. Participants

A total of five legally licensed drivers (2 males and 3 females) participated in the experiment. Their average age was 32 years old (range of 20–45 years). They had held a driver’s license for a mean period of 12.6 years (range of 2–25 years). Before collecting data, we explained the contents of the experiment. In addition, we obtained informed consent from each subject to use the collected data for research purposes.

### 3.2. Experimental Design and Procedures

**Figure 4:**
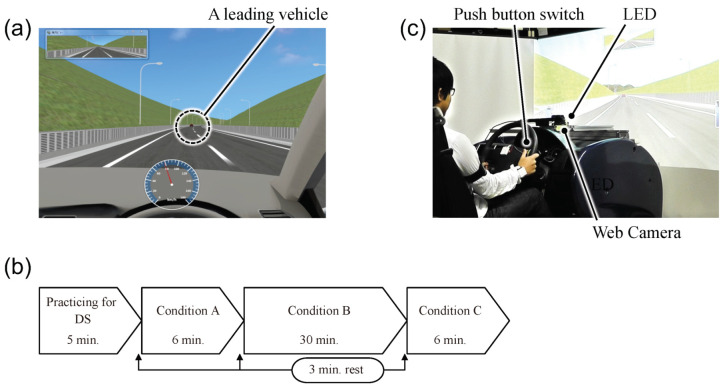
Experimental setup using the driving simulator: (**a**) a course scene on driving simulator; (**b**) driving scenarios; (**c**) layout of LED, push button switch, and web camera.

A relatively simple and monotonous driving task was used to simulate a situation in which the driver’s performance had deteriorated. The driving scenario consisted of a course that simulated a two-lane highway with an eight-shaped loop of approximately 30 km in one time around. Participants were instructed to follow a leading vehicle while maintaining a safe distance but not to change lanes or overtake the leading vehicle. The speed of the leading vehicle was set as 80 km/h. Figure 4a shows a part of the course scene on the driving simulator, which is designed by using UC-Win/Road (FORUM8 Co., Ltd., Tokyo, Japan).

The experiments were conducted in the order shown in Figure 4b while changing the traveling time and traffic situation around the participant’s vehicle. After setting up body-worn sensors, participants were instructed to drive approximately 5 min to allow participants to become familiar with the driving simulator. Then, to obtain the baseline for participants, we set Conditions A and C to drive for approximately 6 min on the course to simulate normal driving. In the condition B, each participant drove for approximately 30 min on the course to simulate the situation of inattentive driving.

To obtain more obvious changes in sleepiness level, all experiments lasted from 2:00 p.m. to 6:00 p.m. In addition, all participants were asked to avoid drinking alcohol and drinks, including caffeine, a day before the experiment.

### 3.3. Annotation of Driver State

To evaluate the drowsiness level, the face image data were captured by a USB webcam installed on the dashboard shown in Figure 4c. The captured images were observed by three trained human referees and were evaluated for the drowsiness level based on the participant’s facial expressions and gestures on a 6-point scale at 5 s intervals, referring to the Ref. [37]. Then, the average value provided by the three referees was calculated. From the results, an interval with an average value of 2.0 or more was defined as “a drowsiness state”, and the other intervals were defined as a “non-drowsiness state”.

To evaluate the inattentive level, the simple reaction time (RT) was measured by the following vigilance task. Participants were instructed to push the button installed on the steering wheel shown in Figure 4c as quickly as possible when they noticed the LED light was illuminated. The LED light installed on the dashboard was programmed to turn on for 2 s at random intervals within 10 s. RTs were calculated from the onset of the LED lights to the participant’s button press. From the results, an interval with the RTs of mean + 1SD or more in each participant was defined as “an inattentive state”, and the other intervals were defined as a “non-inattentive state”.

### 3.4. Data Acquisition

**Figure 5:**
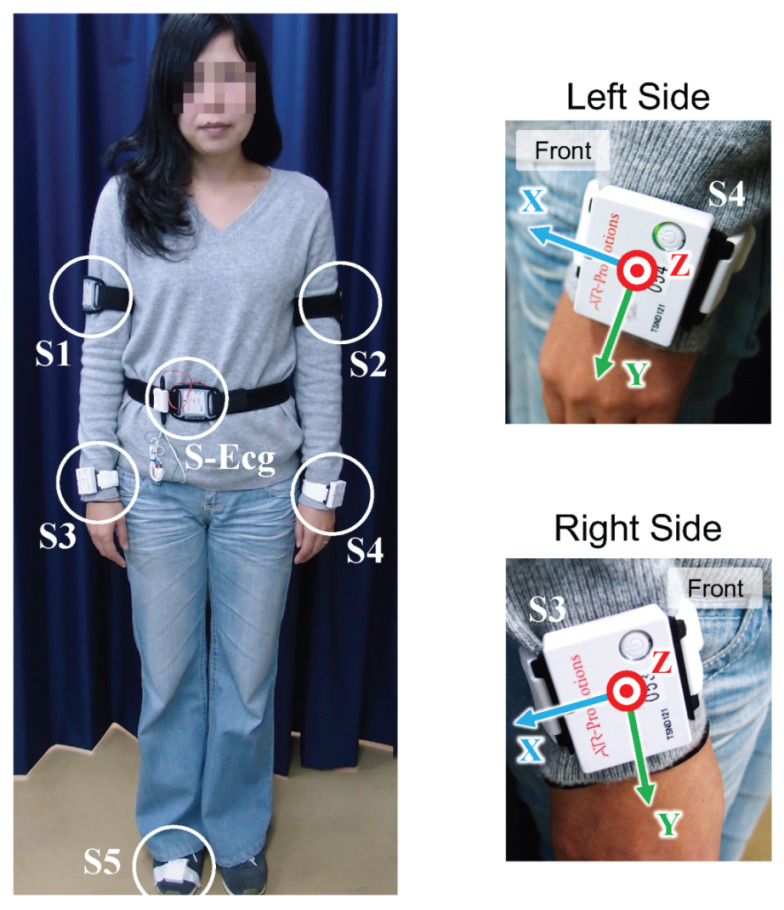
Sensor layout for measuring the body movement of limbs, including wrists and ECG on the chest.

We used a 6-axis inertial sensor (ATR-Promotions Inc., TSND121 [38]) to record the acceleration and gyroscope signals from the body movement of a driver while driving. Five inertial sensors were attached to the driver’s body segments, and their locations and number are shown in Figure 5. These measured signals were sampled at 100 Hz in each sensor module and transmitted to the host computer via Bluetooth. To remove high-frequency noise, all signals were filtered by a third-order Butterworth Low Pass filter with a cut-off frequency of 12.5 Hz.

In addition, to detect driver drowsiness, electrocardiogram (ECG) data were recorded synchronously with the inertial sensor data by using an ECG amplifier module [38]. The ECG signal was sampled at 1 kHz and transmitted to the host computer via Bluetooth. After that, the RR interval (RRI) was extracted from the ECG signal. Since raw RRI data were not sampled at equal intervals, it was interpolated by using spline and resampled at one-second intervals for frequency analysis. Note that, in this study, we measured ECG data on the chests of participants. We could also acquire heart rate data by using wristwatch-type devices, such as the Apple Watch [39].

## 4. Results and Discussions

As a feasibility study, the two detection models separately, excluding the body movement detection model shown in Figure 2a, were constructed by the MSPC method, and their detection rates were verified. In the following, to construct each MSPC model, the dataset collected under condition A described in Section 3.2 was used as a dataset for the normal state. Then, the dataset collected under condition B was used for evaluating the detection accuracy of the constructed models. However, with participants #2 and #4, the dataset of condition C was used for model construction instead of condition A because the datasets of condition A in these participants had missing values due to a problem in ECG measurement. In addition, the MSPC models were constructed and evaluated for each participant using their dataset. In other words, it is a user-dependent model.

Only one principal component was adopted in all MSPC models. The control limit of $T^{2}$ statistics was set so that 90% of the samples used for model construction were below the control limit, and the other 10% above the control limit.

### 4.1. Drowsiness Detection Model

The RRI data were sliced by the sliding window, and eight HRV features described in Section 2.2.1 were calculated within each subsequence. Here, the window size was 120 s and the sliding interval was 1 s. In addition, the autoregressive model using the Yule-Walker method with an order of 10 was used for calculating the PSD of RRI data, referring to [15]. Then, using the procedure provided in Section 2.3, a drowsiness detection model was constructed for each participant of the experiment, where P=8 and $N=N_{\mathrm{drw}}$. Here, $N_{\mathrm{drw}}$ represents the number of subsequences measured since the vehicle started running on the specified course under condition A. The average of $N_{\mathrm{drw}}$ for five participants was 191 subsequences.

### 4.2. Inattentive Detection Model

The accelerometers data from the wrists (S3 and S4 shown in Figure 5), which was selected taking practicality into consideration, were used for the model construction of inattentive detection. The accelerometers data were sliced by the sliding window, and motion features described in Section 2.2.2 were calculated within each subsequence. The three motion features, variance/skewness/kurtosis of the difference values within the subsequence, were defined experimentally, which provided the best overall estimation. Here, the window size was W=60 s, the sliding interval was 1 s, and the number of sub-window was $N_{\mathrm{sw}}=60$. Here, the posture and movement of the hand holding the steering wheel is different for each individual; however, in this study, to detect fine-grained movement associated with steering operation for each direction of both hands, the motion features in Equation (1) were computed for data of the 3-axis acceleration of both wrists. Furthermore, by building a model for each individual, a model of the normal state reflecting each individual’s driving style was constructed. Thus, using the procedure provided in Section 2.3, an inattentive detection model was constructed for each participant of the experiment, where P=18 (2 sensors ×3 axis ×3 motion features) and $N=N_{\mathrm{atv}}$. Here $N_{\mathrm{atv}}$ represents the number of subsequences measured since the vehicle started running on the specified course under condition A. The average of $N_{\mathrm{atv}}$ for five participants was 251 subsequences.

### 4.3. Detection Results

Table 1 shows the detection results of the constructed drowsiness and inattentive detection models for each participant. These sensitivities and specificities were obtained from the datasets in condition B for each participant. The average sensitivity of the drowsiness and inattentive states, which mean the drowsiness and inattentive detection rates, was 52% and 71%, respectively. Here, participant #3 was excluded from the evaluation of drowsiness detection because the facial expression could not be evaluated because of a problem with the captured face images, and consequently, the drowsiness level could not be evaluated.

**Figure 6:**
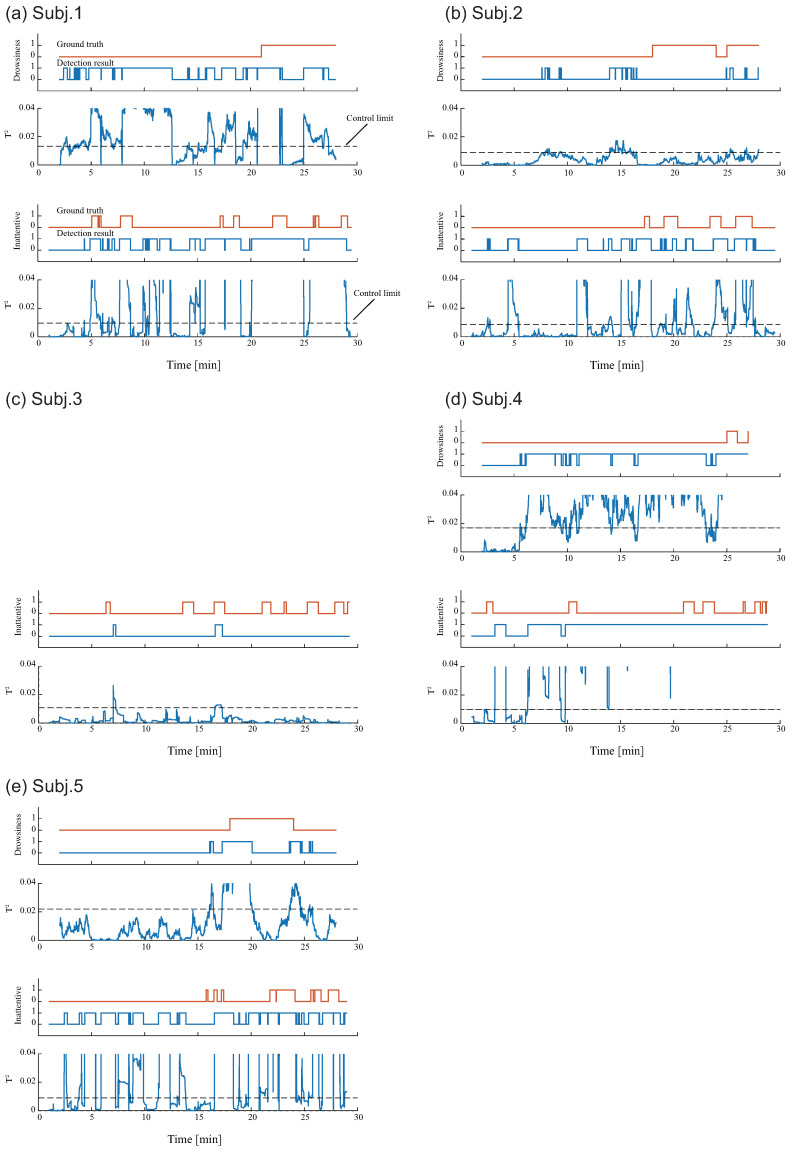
Detection result for five participants: (**a**–**e**) correspond to Subj. 1, 2, 3, 4 and 5, respectively. At each figure, the upper two graphs show the results for the drowsiness detection model; The lower two graphs show the results for the inattentive detection model. The ground truth (Orange line), detection result (Blue line), and the time change of the $T^{2}$ statistic are shown in each graph.

Figure 6a–e shows detection results of drowsiness and inattentive states, respectively, for five participants: the graph of time change of actual state defined by the method provided in Section 3.3 (top); the estimation result of detection model in which a high level is defined when the $T^{2}$ statistics exceed its control limit (center); and the time change of $T^{2}$ statistics (bottom).

### 4.4. Discussion

The drowsiness detection rate obtained in this study was 52%, which is lower than the results shown by Abe et al., whose drowsiness detection rate was 68% [15]. The reasons for this may be due to the missing rate of the RRI data. The average missing rate of the RRI data in this study was 17%, which is a higher rate than the prior research, whose missing rates were less than 2% for all participants. Thus, the obtained results in this study could be improved by detecting the RRIs stably by suppressing the effects of background noise or motion artifacts. On the other hand, Lee et al. [40] used a recurrence plot of ECG data and a convolutional neural network (CNN) to detect drowsy driving. Iwamoto et al. [17] reported that the detection accuracy could be improved by using long short-term memory (LSTM) and raw RRI data instead of conventional HRV features. Although a large enough dataset is needed for the training of these models, further investigation and comparisons of these deep learning-based detection methods are needed in future work.

Further, the inattentive detection rate in this study was 71%, and the sensitivity of three of the participants exceeded 87%, whereas that of one participant was less than 12%. This means that the control limits should be adopted according to each participant. To construct an optimal detection model with the highest sensitivity, it is necessary to choose a personalized control limit for each detection model. To solve this problem, for example, the receiver operating characteristic curves based on the signal detection theory will be used. In the inattentive detection model, the false positive rate was 42.5%. The reasons for this include the fact that when the body movement of the driver occurred, the motion features notably fluctuated, which were determined as anomalies by the inattentive detection model. This means that the body movement detection model can detect movements not related to driving, and may play a role in improving the false positive rate. Further studies will investigate and verify a design that integrates the three detection models described in Section 2.1. On the other hand, Kume et al. [3] showed the specificity of inattentive state was 87% (i.e., the false-positive rate was 13%) with a manually set threshold by using data of steering angle and vehicle speed. Note that in their study, the inattentive state was defined by subjective evaluation and predicated at 3 min intervals. Moreover, we constructed and evaluated the inattentive detection model using sensor data from both wrists. However, the tendency of change in the motion features could also be affected by the difference in traffic rules of the country or region, for example, left-/right-hand drive. Thus, further investigation of the invariant features that does not depend on the usage environment or individual is needed. Another issue is how to set the control limit in consideration of individual differences.

**Figure 7:**
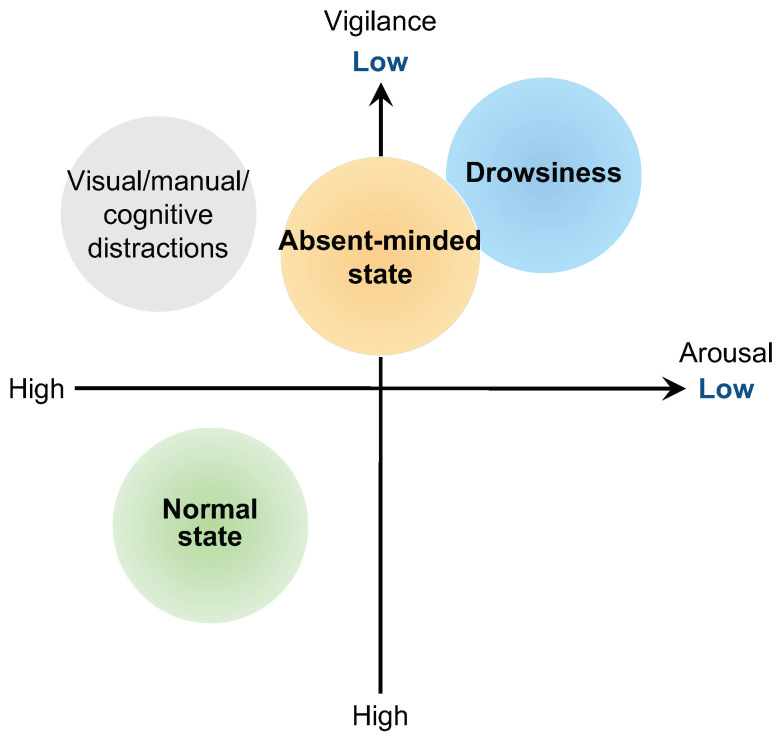
An overview of the categories of the driver’s internal state. Note that the categories in this graph correspond to Table 2. Also, the study of drowsiness driving using body-worn sensors, which are referred to in this paper, can be found in the literature [15,17,23,40], the study of distracted driving, in [24,25,26], and the study of driving in the absent-minded state, in [3] and this study.

Here, we summarize the comparison with other approaches, which are referred to by this paper, in Table 2. Additionally, an overview of the categories of the driver’s internal state shown in Table 2 is summarized in Figure 7. Table 2 and Figure 7 show that similar studies for detecting inattentive driving in an absent-minded state are still very limited, compared to drowsiness and distraction detection. Moreover, from “ground truth” in Table 2, we see that the definition of driver’s internal state is one of the most important issues because the standard labeling method has not yet been developed. Additionally, the experimental conditions, such as scenario and platform, are different for each study. Because of that, it is immediately more difficult to discuss and compare the results for each study directly.

## 5. Conclusions

In this study, we proposed an algorithm for detecting inattentive states and drowsiness, which occurs in long, monotonous driving scenarios, such as highways, using body-worn accelerometers and a heart rate sensor. The proposed method consists of three detection models, that is, body movement, drowsiness, and inattention detection based on an anomaly detection algorithm. We also reported on the evaluation results of two of these detection models: drowsiness, and inattentive detection. In our approach, to detect inattentive driving, motion features extracted from the acceleration signals on wrists were used. Additionally, to detect drowsiness driving, HRV features extracted from RRI data were used. Each detection model was constructed on the basis of an anomaly detection algorithm using MSPC. Our approach and its verification result indicate the feasibility of driver monitoring via wrist-worn sensors. Moreover, since our method can be realized by using wearable sensor devices, a driver’s state can be measured without dependence on the type of vehicle. On the other hand, in non-monotonous driving scenarios, our algorithm could be challenging to apply because there is a large variability in motion features of the wrists associated with steering operations of overtaking or right/left turn.

The results of this study were from a limited number of participants. According to NHTSA guidelines [41], further investigation of the effect of detecting a driver’s internal state for a wide range of age groups is required. Moreover, in the experimental design of this study, the amount of usable data is limited for building and verifying the user-dependent models; that is, there are only three sessions of data for each participant. Therefore, improving the experimental design, such as increasing the session, is needed to construct and expand the dataset. In future work, we will integrate the detection models and expand the size of the dataset for verification, thus scaling our approach for real-car usage.

## Figures and Tables

**Table 1:** Detection accuracy for all participants.

	Drowsiness		Inattention	
**Subj.**	**Sensitivity**	**Specificity**	**Sensitivity**	**Specificity**
1	0.59	0.33	0.94	0.47
2	0.06	0.86	0.73	0.75
3	-	-	0.12	0.99
4	1.00	0.26	0.87	0.17
5	0.41	0.91	0.91	0.49
Avg.	0.52	0.59	0.71	0.58

**Table 2:** Comparison table of related studies for detecting a driver’s internal state using wearable-type sensors. Note that the exception is that Kume et al. [3] is an in-vehicle sensor-based method. Additionally, n/a means that no description for details can be found.

Study	Category	Measuring Method	Participant# (Male:Female, Age)	Scenario	Platform	Ground Truth
Abe et al. (2016) [15]	Drowsiness	Wearable RRI telemetry	27 (17:10, 20 s to 40 s)	Driving on a highway loop line at night for two hours	DS	Facial expression rating by human referees
Lee et al. (2019) [40]	Drowsiness	Wristwatch-type PPG and Chest-belt-type ECG sensor	6 (n/a, 20 to 35)	n/a	DS	Visual evaluation of facial and body movement
Iwamoto et al. (2021) [17]	Drowsiness	ECG with chest electrode	25 (17:8, mean 21±1.8)	A monotonous driving task in a dark room for three hours	DS	Labeled based on sleep specialist’s score
Lee et al. (2015) [23]	Drowsiness	Wristwatch-type PPG and Wrist-worn IMU sensors	12 (9:3, 21 to 45)	Highway driving simulation	DS	Karolinska sleepiness scale (KSS) every 2 min
Jiang et al. (2018) [24]	Manual distraction	Wrist-worn IMU sensor (on the right wrist)	20 (10:10, 25 to 35)	Participants perform five different hand gestures, such as smartphone use	Real	Manually labeled
Tanaka et al. (2020) [25]	Cognitive distraction	Wrist-worn IMU sensors	7 (7:0, mean 22±1.5)	A monotonous driving task with a cognitive task called N-back task	DS	The task level, that is, N in the N-back task
Sun et al. (2021) [26]	Manual distraction	Wrist-worn IMU sensor (on the right wrist)	20 (14:6, 21 to 35)	Participants perform four types of gestures; three manual distractions and one regular driving motion	Real	Manually labeling by a passenger
Kume et al. (2014) [3]	Drowsiness and absentminded state	Steering wheel angles and vehicle speed	34 (16:18, 20 s to 60 s)	Driving for 1.5 h on the specified highway section	Real	Subjective evaluation on a 5-point scale per 3 min
This study	Drowsiness and absentminded state	Wrist-worn IMU sensors and ECG with chest electrode	5 (2:3, 20 to 45)	A monotonous driving task for approximately an hour	DS	Facial expression rating and reaction time (see Section 3.3)

DS: driving simulator, Real: real vehicle platform, IMU: inertial measurement unit, PPG: photoplethysmogram.

## Data Availability

All data in this research are available upon request to T. Akiduki.
